# Nutrition and Health in the Lesbian, Gay, Bisexual, Transgender, Queer/Questioning Community: A Narrative Review

**DOI:** 10.1016/j.advnut.2023.07.009

**Published:** 2023-08-02

**Authors:** Elisabetta M. Ferrero, Alexandra G. Yunker, Sherri Cuffe, Saloni Gautam, Kenny Mendoza, Shilpa N. Bhupathiraju, Josiemer Mattei

**Affiliations:** 1 Department of Nutrition, Harvard T.H. Chan School of Public Health, Boston, MA, United States; 2 Channing Division of Network Medicine, Brigham and Women’s Hospital and Harvard Medical School, Boston, MA, United States

**Keywords:** LGBTQ+, nutrition, sexual and gender minorities, food insecurity, disordered eating, mental health

## Abstract

Sexual and gender minorities have a higher risk for health and nutrition-related disparities across the life course compared to the heterosexual or cisgender population. Experiences of stigmatization and discrimination are associated with diminished mental health quality and psychological distress, which are risk factors for developing various eating disorders. Other nutrition disparities include increased risk for food insecurity, body dissatisfaction, and weight complications, such as those experienced by the transgender population in association with gender-affirming hormone therapies. Despite the need for tailored nutrition recommendations that address the unique needs of the lesbian, gay, bisexual, transgender, queer/questioning (LGBTQ+) community, there are currently no such guidelines in North America. The purpose of this review is to summarize major LGBTQ+ nutrition disparities and highlight the need for tailored recommendations. We examine the evidence on mental health and social disparities in this group, including vulnerabilities to disordered eating, food insecurity, and healthcare provider discrimination. Importantly, we identify a scarcity of literature on dietary concerns and nutrition care guidelines for LGBTQ+ groups, including studies that address intersectionality and differences among specific gender and sexual orientations. These gaps underline the urgency of prioritizing nutrition for LGBTQ+ health needs and for developing tailored public health nutrition recommendations for this underserved population. Our review suggests that future LGBTQ+ health and nutrition research agendas should include personalized and precision nutrition, social determinants of health, diet quality, body image, and healthcare provider cultural competency and responsiveness. Moreover, the current evidence on LGBTQ+ nutrition and health will be strengthened when research studies (including clinical trials) with robust methodologies amplify inclusion and representation of this community to elucidate health and nutrition disparities in sexual and gender minorities.

**Statements of significance**

This review contributes summarized evidence on several public health nutrition considerations among the LGBTQ+ community, highlighting dietary, behavioral, social, and system-wide vulnerabilities that increase health disparities across and within specific groups. The review reveals a scarcity of comprehensive literature on this topic that generally combines sexual and gender minorities into 1 group, neglecting their unique attributes and needs. Increasing statistics and awareness of LGBTQ+ communities require immediate attention to their nutrition and health, including tailored recommendations by group, underscoring the significance of this review.

## Introduction

**FIGURE 1:**
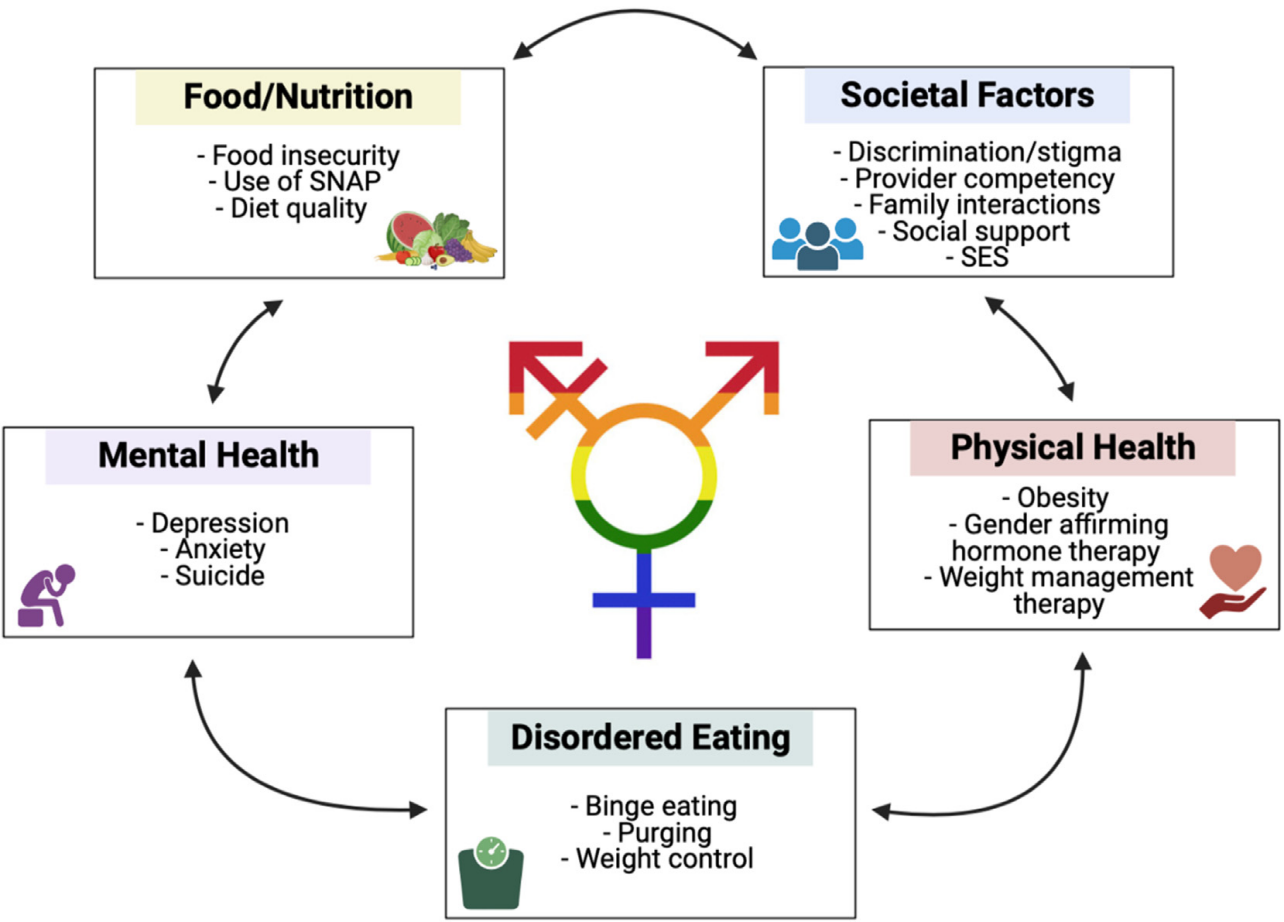
Conceptual model of the interrelation between food/nutrition factors, societal factors, behaviors, and health in the LGBTQ+ community. Created using BioRender. LGBTQ+: lesbian, gay, bisexual, transgender, queer/questioning; SES: socio-economic status; SNAP: supplemental nutrition assistance program.

The lesbian, gay, bisexual, transgender, queer/questioning (LGBTQ+) community consists of a cross-cultural group of people of distinct sexual orientations and gender identity groups, including all races, ethnic and religious backgrounds, and socio-economic statuses [1]. Compared to the heterosexual population, LGBTQ+ individuals experience significant health disparities across the life course, including marked occurrence of HIV or other sexually transmitted diseases, psychiatric disorders, substance abuse, and suicide [2]. Experiences of homophobia, stigmatization, and marginalization elevate distress, limit coping strategies, contribute to depression, and interfere with health and well-being [3]. Additionally, the LGBTQ+ community experiences nutrition-related disparities in areas of obesity, eating disorders, body dissatisfaction, and food insecurity [4] and faces challenges in receiving individualized clinical nutrition counseling and treatments due to lack of training, cultural competency and responsiveness among healthcare providers and services [5]. Studies have highlighted concerns regarding eating disorders and body dissatisfaction, especially among gay men [6]. Other studies examining weight disparities across sexual orientations found that lesbian women are more likely to be overweight than heterosexual women [7]. Our conceptual model illustrates some of the salient nutrition and mental health disparities in the LGBTQ+ population and highlights their interconnection with societal factors, such as discrimination and stigma (Figure 1).

Despite the evidence, the literature on LGBTQ+ nutrition is extremely limited and does not provide nutrition advice tailored to the community. Similarly, the current Dietary Guidelines for Americans [8] do not provide any recommendations for LGBTQ+ individuals. Additionally, there are significant differences in health behaviors across LGBTQ+ groups, highlighting the importance of investigating different dietary needs for populations that have been historically grouped into 1 [6]. For example, the transgender population needs particular attention in clinical nutrition, given the complexity of medical interventions involved in facilitating gender-affirming therapies. Therapies such as gender-affirming hormone therapy may cause endocrine and cardiometabolic changes, such as changes in lipid profile that should be addressed through tailored nutritional interventions [9].

In 2023, 9% of adults across 30 countries, as well as in the United States, considered themselves to be lesbian, gay, bisexual, transgender, or any other sexual and/or gender orientation other than heterosexual and/or cisgender, with twice that proportion (18%) among Generation Z members (born after 1997) [10]. Thus, it is critical to direct the attention of the nutrition community to LGBTQ+ needs. The objective of this narrative review was to provide a comprehensive overview of main nutrition-related disparities among the LGBTQ+ population in North America and their exacerbating effects on LGBTQ+ vulnerabilities, following our conceptual model. We summarized main studies on mental health, eating disorders, food insecurity, poor diet quality, and distinctive risks for the transgender community (Table 1). The review highlights the need to prioritize LGBTQ+ nutrition on the public health agenda and to provide tailored and culturally responsive nutrition guidelines and programs that account for within groups variability. The terminology used in the paper includes LGBTQ+, LGBTQ, LGBT, and LGB in order to be consistent with the literature when reporting results about specific groups and be respectful of the differences among populations.

**TABLE 1:** Characteristics of main nutrition-related studies cited in the narrative review

First author (reference)	Sample size and sex and/or gender	Design, year, and setting	Main results
**Eating disorders**			
Bell [17]	Total *n* = 317 *n* = 97 gay men *n* = 82 lesbian women *n* = 138 TGNC	Cross-sectional study. Participants from a 2017 United States national study conducted by the Laboratory for Resilience in Psychological and Physical Health.	Eating disorders were higher in lesbians (66.7%), followed by TGNC (62.6%) and gay men (47.6%). Higher odds of eating disorders were noted for higher depression in lesbians, lower self-compassion in TGNC, and higher depression and perceived stigma and lower self-compassion in gay men.
Hazzard [18]	Total *n* = 118,421 *n* = 17,933 sexual minorities	Cross-sectional study of cisgender men and women from 178 United States colleges and universities participating in the Healthy Minds Study between 2016 and 2019.	Higher odds of eating disorders were noted for male participants who identified as questioning (OR = 2.57; 95% CI: 1.88, 3.51) or bisexual (2.22; 95% CI: 1.84, 2.67) and females who identified as questioning (1.39; 95% CI: 1.24, 1.56) or bisexual (1.40; 95% CI: 1.31, 1.50) compared to heterosexual counterparts, and underdiagnosis relative to gay or lesbian peers.
Austin [27]	*n* = 13,795	Prospective cohort study. Youth ages 12–23 y. Data from GUTS study on United States youth. Questionnaires were administered in 1996, 1998–2001, 2003, and 2005.	Females who identified as bisexual had higher odds of reporting purging behaviors compared to their “mostly heterosexual” (OR = 1.4; 95% CI: 1.0, 1.9) and lesbian (2.1; 95% CI: 1.0, 4.2) counterparts. Among males, all 3 sexual orientation groups were more likely than heterosexual males to report both binge eating and purging.
Watson [30]	*n* = 923	Cross-sectional study. Transgender youth, ages 14–25 y from the 2014 Canadian Trans Youth Health Study.	Harassment and discrimination were associated with higher odds of past year binge eating (OR = 1.06; 95% CI: 1.01, 1.12), fasting (1.10; 95% CI: 1.05, 1.16), or vomiting to lose weight (1.07; 95% CI: 1.01, 1.13). Protective factors (i.e., family connectedness, school connectedness, caring friends, and social support) were linked to lower odds of disordered eating.
**Diet quality**			
Prestemon [40]	Total *n* = 8851 *n* = 4230 heterosexual men *n* = 173 gay or bisexual men *n* = 4165 heterosexual women *n* = 283 lesbian or bisexual women	Cross-sectional study. Adults aged 20–65y participating in the United States 2011–2016 NHANES.	Dietary outcomes (daily EIs of 20 food/beverage groups and the total Healthy Eating Index score) for gay, lesbian, and bisexual participants were similar to outcomes of their heterosexual counterparts. Gay and bisexual males had a healthier intake of processed meats and higher overall dietary quality compared to heterosexual males (53.40 ± 1.36 vs. 49.29 ± 0.32, respectively).
**Food security**			
Arikawa [16]	*n* = 253	Cross-sectional study. Participants identifying as LGBTQ+, ages 18–35 y. Data were collected between March 2018 and March 2019.	The proportion of members of the LGBTQ+ community who reported experiencing food insecurity was 54.4%, and food insecurity was particularly prevalent among individuals who identified as trans males (frequency 64.8%).
Patterson [37]	Total *n* = 7379 *n* = 88 lesbian *n* = 251 bisexual *n* = 366 heterosexual and reporting same-sex behavior *n* = 6674 heterosexual	Cross-sectional study. Women who participated in the United States 2004-2014 NHANES.	All sexual minority women reported a higher prevalence of food insecurity over the past 12 mo than heterosexual women (20.6–27.3% vs. 13.1%). Lesbians and women reporting same-sex behaviors were more likely to rely on emergency food assistance [range of prevalence ratios = 1.34; 95% CI: 1.05, 1.70 to 1.84, 95% CI: 1.13, 3.01].
Brown [38]	-	Cross-sectional study of persons identifying as LGB/T using Multiple United States surveys, including NHIS and NSFG. Data were collected between 2011–2014.	Food insecurity and SNAP participation were more common among LGB/T individuals (27% and 27%, respectively) than their non-LGB/T counterparts (17% and 20%, respectively).
Russomanno [49]	*n* = 20	Qualitative study. Structured telephone interviews were conducted between April–June 2017 with transgender and gender non-conforming individuals in the Southeast United States.	Participants reported living in extreme poverty and facing substantial barriers, such as unemployment and discrimination. Poverty, food insecurity, and unemployment are serious concerns for transgender and gender-non-conforming individuals, and they erode their physical and mental health, as well as their support systems.
**Obesity**			
Laska [4]	Total *n* = 33,907 *n* = 12,498 men *n* = 21,384 women	Cross-sectional study. Students of 40 2- and 4-y colleges and universities in Minnesota participated in a state-based survey in 2007–2011.	Bisexual and lesbian women as more likely to be classified as obese than heterosexual women. Gay and bisexual men exhibited poor activity patterns; however, men who identified as gay consumed fewer sugar-sweetened beverages and non-nutritive sweetener drinks compared to males who identified as heterosexual.
Austin [28]	Total *n* = 13,785 *n* = 9039 girls *n* = 7843 boys	Prospective cohort study. Youth ages 12–23 y, part of the GUTS cohort. Questionnaires were administered in 1996, 1998–2001, 2003, and 2005.	Among females, BMI was consistently elevated in all sexual orientation minorities compared to their heterosexual counterparts. Among males, a sexual-orientation-by-age interaction was found, with steeper increases in BMI with age from early to late adolescence in heterosexuals compared to sexual orientation minorities.
Azagba [31]	Total *n* = 716,609 *n* = 685,096 heterosexual *n* = 10,353 gay or lesbian *n* = 11,255 bisexual *n* = 9905 unsure	Cross-sectional study. Data from 2014–2017 BRFSS surveys in the United States.	Compared to their heterosexual counterparts, lesbian and bisexual women were more likely to be classified as overweight (OR = 1.33; 95% CI: 1.17, 1.53 and 1.21; 95% CI: 1.10, 1.34, respectively) or obesity (1.49; 95% CI: 1.31, 1.70 and OR = 1.43; 95% CI: 1.29, 1.59, respectively). Compared to their heterosexual counterparts, gay men were less likely to be classified as overweight (OR = 0.66; 95% CI: 0.59, 0.73) or obese (OR = 0.77; 95% CI: 0.69, 0.86). There were no significant differences for bisexual men.
Stupplebeen [39]	Total *n* = 72,214 *n* = 69,299 heterosexual *n* = 2915 gay/bisexual	Cross-sectional study. Data from male adult participants in the 2003–2012 California Health Interview Survey.	Weight status was positively associated with hypertension, asthma, heart disease, and diabetes in gay, bisexual, and heterosexual men. Among gay and bisexual men, the associations were stronger and statistically significant.
Kyinn [56]	Total *n* = 470 *n* = 247 transfeminine *n* = 223 transmasculine	Longitudinal prospective study. Transgender and gender-diverse adult patients seen at a Federally Qualified Health Center and an academic endocrinology practice, both in Washington DC, United States; data collected between 2007 and 2015.	As compared to transfeminine individuals, transmasculine individuals had higher rates of weight gain and obesity before and during gender-affirming hormone therapy.
van Velzen [57]	Total *n* = 430 *n* = 242 transwomen *n* = 188 transmen	Prospective cohort study. Substudy of the European Network for the Investigation of Gender Incongruence. Measurements were taken before and after 12 mo of hormone therapy; 2010–2017.	Hormone therapy effects on blood pressure were negligible, but unfavorable changes in lipid profile were observed in transmen, and a favorable effect was noted in transwomen.
Shadid [58]	Total *n* = 90 *n* = 35 transgender men *n* = 55 transgender women	Prospective cohort study. Substudy of the European Network for the Investigation of Gender Incongruence. Body composition and OGTT were evaluated before and after 1 y of gender-affirming hormone therapy; 2010–2014.	Insulin sensitivity and post-OGTT incretin responses tended to increase with masculinization and decrease with feminization.
Klaver [60]	Total *n* = 341 *n* = 179 trans women *n* = 162 trans men	Prospective observational cohort; 2010–2015. Changes in total body and visceral fat were measured before and after 1 y of gender-affirming hormone therapy.	Hormone therapy in trans women and trans men resulted in changes in ratio of visceral fat to total body fat, mainly because of changes in total body fat, and were unrelated to changes in cardiometabolic risk factors.

GUTS: Growing Up Today Study; LGBTQ+: lesbian, gay, bisexual, transgender, queer/questioning; NSFG: National Survey of Family Growth; SNAP: Supplemental Nutrition Assistance Program; TGNC: transgender and gender non-conforming; BRFSS: Behavioral Risk Factor Surveillance System.

## Mental health

Historically, public health promotion for the LGBT community has focused on safe sexual practices, if any attention was given at all. While such concerns around physical health disparities are justified, the LGBT community is more than twice as likely to have a mental health disorder in their lifetime [11] and 2.5 times more likely to experience depression, anxiety, and substance misuse, compared with heterosexual individuals [12]. Furthermore, evidence suggests that LGBT individuals are more than twice at risk of suicide attempts compared to heterosexual individuals (OR = 2.47; 95% CI: 1.87, 3.28). The rate of suicide attempts is 4 times greater for lesbian, gay, and bisexual youth than that for heterosexual youth [13].

Among the LGBT community, discrimination, homophobia, and stigma are all associated with diminished mental health quality and higher risk for psychological stress, depression, and anxiety [14]. This phenomenon has been documented and explained through the minority stress model, which posits that sexual minorities experience unique and hostile stressors in relation to their sexual minority identity and consequently suffer from negative health effects [15].

These mental health disparities are risk factors for the development of eating disorders and behaviors associated with weight control, such as restrictive dieting, binge eating, and purging [16], and require immediate public health attention. It has been found that sexual minorities who report a higher level of stigmatization are more likely to report eating disorder symptoms [17]. Additionally, norms concerning appearance among sexual minorities generally play a critical role in development of eating disorders [18].

### Social support and protective factors

A limited scientific research literature exists on the protective social factors for LGBT youth and young adults, including family relationships [19]. However, evidence does suggest LGBTQ individuals may have less social support than heterosexual individuals, particularly if they live in a region without a large LGBTQ population or if they have experienced rejection by their family of origin [20].

Ryan et al. [19] conducted a participatory research study on how family interactions influence mental health, substance abuse, and sexual risk of LGBT adolescents and young adults in 245 LGBT Latino and non-Latino individuals from 249 LGBT venues. The study showed that family acceptance is associated with positive health outcomes regarding better self-esteem, social support, and general health, promoting disclosure of nonheteronormative status. In addition, family acceptance is protective against depression, substance abuse, and suicidal ideation and attempts [19]. Other forms of support include support from peers and significant others, although family support seems to be more protective than peer or significant-other support for non-suicidal self-injury, alcohol use among adolescents [21], and school performance among multi-ethnic sexual minority youth [22]. Because social support is critical in reducing psychological distress among sexual minorities, it can be a protective factor against eating disorders.

The level and nature of support received might differ based on individual characteristics, such as birth sex, socio-economic status (SES), or sexual orientation. Results of a study suggest that, compared to LGBT youth assigned female at birth, LGBT youth assigned male at birth are more likely to struggle with developing supportive relationships with peers and significant others, perhaps in association with traditional masculinity rules that may discourage the development of healthy support systems for LGBT assigned male at birth [23]. Results for SES show that youth with low SES are less likely to receive family support than high SES youth [23]. Additionally, a study found that, for lesbians, no forms of support are associated with self-esteem [24]. Other research found that immigration status is an additional determinant of marginalization and stigma among LGBT individuals [25]. These differences highlight the need to understand the mechanisms underlying support among the different groups.

### Disordered eating and weight control behaviors

Although there is an abundance of literature demonstrating that disordered eating behaviors in young adults and adolescents can contribute to health complications and negatively impact overall quality of life and mortality [26], there are only a small number of studies investigating disordered eating behaviors specifically within the LGBT community. Of note, Austin et al. [27] used data from the Growing up Today Study, a large prospective cohort of United States youth, to describe sexual orientation and weight status trajectories among adolescents. Using the Growing up Today Study, Austin et al. [27] investigated purging and binge eating trends from early through late adolescence (12–23 y) in female and male youth across sexual orientations [28]. The study reported that bisexual adolescents are twice as likely to binge eat (OR = 2.2; 95% CI: 1.6, 2.9) and to purge (OR = 2.2; 95% CI: 1.6, 3.2) compared to their heterosexual peers [28]. In addition, 20% of gay and bisexual high school males report disordered weight control behaviors (such as restricting, purging, and using diet pills), compared to only 5% of heterosexual male peers [27]. Moreover, LGBTQ youth begin displaying disordered eating behaviors (including binge eating and/or purging) as early as 12–14 y of age, which often persists into adulthood [29]. Of note, additional studies in adults show that bisexual and gay men are more likely to binge compared to heterosexual men (18.7% and 22.1% compared with 11.4%; *P* < 0.0001) [4,30].

Previous equivocal findings regarding body weight disparities between heterosexual and sexual minority populations may be partly explained by differences in rates of overweight/obesity between sexual minority subgroups [31,32]. For example, while heterosexual males experience a steeper BMI kg/m^2^ increase from early to late adolescence compared to their sexual minority counterparts; conversely, sexual minority female adolescents have elevated BMI compared to their heterosexual peers [28]. Similarly, bisexual (12.6%) and gay (15.5%) adult women are significantly more likely to have obesity compared to heterosexual women (9.8%). These collective data suggest that in addition to health inequities between heterosexual and LGBTQ populations, there are also alarming within-group disparities for sexual minorities. To help address these disparities in risk for overweight/obesity between LGBTQ subgroups, clinicians and future researchers should utilize an intersectional framework when considering nutrition and weight management strategies for sexual minorities.

## Vulnerability to food insecurity and quality of diet

The USDA defines food insecurity as having limited access to adequate food because of lack of money and/or other resources [33]. Food insecurity is associated with multiple leading causes of death and disability, including cancer, chronic obstructive pulmonary disease, stroke, and diabetes [34], in addition to associated health risk factors (e.g., poor nutrition, obesity, smoking, and chronic inflammation) [35]. In the United States, food insecurity is predominantly measured through the Food Security Supplement and Current Population Survey[36]. Although the current population survey provides important information regarding race, ethnicity, sex, and age, it does not account for a respondent’s sexual orientation or gender identity. Consequently, there is a lack of reliable data to examine food insecurity among most LGBTQ-headed households. Despite this, a small body of research indicates that the LGBTQ community experiences a higher prevalence of food insecurity and poverty than heterosexual individuals [37,38] and disparities in diet-related diseases. For example, a study found that weight status was positively associated with hypertension, asthma, heart disease, and diabetes in gay, bisexual, and heterosexual men, but the associations were stronger among gay and bisexual men [39].

However, some research suggests gay and bisexual males display better overall dietary quality than heterosexual males, specifically in relation to FA and sodium scores. Gay and bisexual males also report lower percent EI of red and processed meat and other animal-based foods compared to heterosexual males, which may be indicative of better diet quality and lower diet-related disease risk. LGBTQ individuals consumed high-caloric beverages, although at a lower rate compared to their straight peers (gay = 39.0%, lesbian = 38.9%, bisexual = 39.2%, queer = 28.7%, and pansexual individuals = 34.1% compared with straight = 47.4%; *P* < 0.0001). There is little evidence of significant differences for other food groups, such as fruits, vegetables, desserts, and sweet snacks [40]. Although the reasons for different dietary intake by gender and sex group warrant more research, some evidence suggests that the noted lower diet quality and higher intake of energy, fats, sodium, and animal products among heterosexual men (and to some extent, the opposite dietary intakes among heterosexual women) may be attributed to perceptions of masculinity and femininity [[41], [42], [43], [44]]. Clinical and public health professionals may leverage these gender-based attitudes for tailored or well-defined diet and health messages for people in the LGBTQ+ gender identity continuum.

Aside from studies on dietary intake, LGBTQ identity and status as a member of the LGBTQ community have been shown to significantly increase the odds of participating in the United States Supplemental Nutrition Assistance Program (SNAP) and overall food insecurity experiences [45]. Brown et al. [38] found that rates of food insecurity and SNAP participation were higher for LGBT adults and same-sex couples when compared to non-LGBT adults or adults in different-sex couples, after taking into account gender, age, racial/ethnicity, and education levels, respectively. As many as 1 in 4 LGBT adults have experienced a time when they did not have enough money to feed themselves or their families, compared to 17% of non-LGBT adults [38]. Of note, data from Brown et al. [38] further indicated that people who identify as bisexual were particularly vulnerable to food insecurity. Furthermore, the COVID-19 pandemic has exacerbated food insecurity among LGBT adults. According to data from the United States Census Bureau, LGBT adults living in the United States were nearly twice as likely to be experiencing food insecurity during the pandemic than non-LGBT adults [46]. Notably, within the LGBTQ community, food insecurity is more common among individuals who are women, members of racial or ethnic minorities, aged 18–49 y, without a college (or higher) degree, unmarried, or with children at home [45].

Most prior studies have focused on the relationship between economic insecurity as a possible pathway to explain high rates of food insecurity in this population, and a nationwide survey in the United States indicates that nearly 1-in-5 LGBT adults reported living in a household with lost employment income in the past 4 wk, compared to just under 17% for non-LGBT adults [46]. In addition, 8% of LGBTQ people were unemployed [46], which was much higher than the national unemployment level (4.1% at the end of 2017) [45]. Badgett et al. [47] and Albelda et al. [48] have also documented higher rates of poverty among LGB people than the rest of the population.

Community and policy-level interventions should be employed to reduce food access disparities faced by the LGBTQ community. At the community level, this may include increasing access to local food assistance for those who do not qualify for SNAP benefits, locally organized food-sharing communities (i.e., via online social platforms), and healthy food prescription programs (i.e., via local non-profit, clinic, and/or larger community health systems initiatives). Most notably, community-level interventions aimed at combating disparities in food access and security should emphasize gender-affirming practices and partner with trusted LGBTQ organizations in order to successfully promote safe and equitable resources for sexual minorities [49]. On the policy level, it is important to address limitations of SNAP benefits that disproportionately disadvantage the LGBTQ community; for example, policymakers should consider expanding SNAP eligibility criteria to exclude work requirements, given that people who identify as sexual minorities face work discrimination that may contribute to higher unemployment rates [50]. Lastly, decreasing food insecurity in the LGBTQ community also requires addressing determinants of economic instability, and this may involve instituting federal and/or state non-discrimination laws that protect sexual minorities from employment discrimination.

## Distinctive risks in the transgender community

Transgender individuals display different risks when studied independently. Compared to the cisgender population, transgender people experience high rates of poverty, joblessness, homelessness, food insecurity [49], and barriers in accessing quality health care [51]. The 2015 United States Transgender Survey showed that 29% of transgender individuals were living in poverty compared to 12% of individuals in the general population, and the unemployment rate among transgender was 15% compared to the 5% United States unemployment rate [52]. Transgender participants in a study repeatedly reported experiencing episodes of stigma and discrimination that affected their financial stability and ability to afford adequate food [49].

As a result of exposure to stress, transgender individuals are at risk of developing disordered eating behaviors, but a review found that this risk might be secondary to the distress that transgender individuals experience in relation to body dissatisfaction [53]. Additionally, there is a high prevalence of self-injurious thoughts in transgender individuals with eating disorders [54].

Cardiometabolic risks and weight management constitute another major challenge for the transgender population in association with gender-affirming hormone therapies (GAHT) [55]. GAHT is a foundational medical therapy for many transgender and gender-diverse individuals. Adult transmasculine individuals are prescribed testosterone, while adult transfeminine individuals are prescribed a combination of estrogen and antiandrogen [56]. These hormone therapies are associated with cardiometabolic risks. A study found that hormone therapy is associated with unfavorable lipid changes in trans men after 1 y of hormone therapy [57]. Another study found that feminizing hormone therapy induces insulin resistance [58].

Additionally, hormone therapy is associated with changes in body composition and weight gain [56]. Weight gain is one of the most common side effects of GAHT, and further research is needed in order to elucidate the mechanism of gender-affirming hormone treatment [55,59]. In a longitudinal study, Kyinn et al. [56] assessed the BMI and overall body weight of 470 transgender and gender-diverse adult patients at baseline and followed them 2–21 mo after starting hormone therapy. Within 2–4 mo of starting hormone therapy, they saw a mean increase of ∼2.35 kg within the transmasculine group [56]. The prevalence of obesity in the transmasculine group increased from 39% at baseline to 42–52% following the initiation of hormone therapy [56]. Alternatively, the transfeminine group was initially stable for the first 21 mo after hormone therapy and began to increase steadily after then [56]. Notably, mean body weight increased the most in those under 30 y of age [56]. The prevalence of obesity in the transfeminine group was 25% at baseline and 21–30% following the initiation of hormone therapy [56]. Both transmasculine and transfeminine groups experienced an increase in body weight and prevalence of obesity rates, but the transmasculine group was most affected by the treatment [56]. The authors concluded that weight-reduction interventions would be appropriate for both groups through dietary intervention [56].

A study suggested that unfavorable changes in cardiometabolic risk factors are directly associated with hormone therapy and not with changes in total body fat among transgender individuals [60]. Nevertheless, changes in body composition pose a serious risk for the transgender population. Obesity and cardiometabolic risk factors can contribute to the development of additional non-communicable diseases such as CVD and type 2 diabetes [61], and it is critical to promote weight-reduction interventions tailored to this group.

Despite the need for tailored interventions, there are significant gaps and lack of literature on clinical nutrition recommendations and nutritional guidelines for individuals undergoing hormone therapy, posing an issue for clinicians who are trying to accurately conduct a gender-affirming nutrition assessment, all while considering whether the patient is experiencing normal or abnormal side effects of the hormonal therapy they are undergoing [61]. For example, a male patient transitioning to a female with a waist circumference of 93.98 cm would be considered healthy for a male circumference (<101.6 cm) but, in turn, would be considered higher than the acceptable healthy waist circumference of a female (>88.9 cm) [62]. This obsolete guide may, in turn, contribute to errors in accurately categorizing a patient’s nutrition and body composition status as either healthy or as having excessive abdominal obesity that may require lifestyle approaches.

Many clinicians will choose to opt out of using these antiquated female or male reference values and personalize the nutrition care based on what stage of transition the patient is in, but this means that they may or may not be able to utilize evidence-based guidelines for their care [62]. This type of care presents a great opportunity to apply precision nutrition by using the patient’s microbiome or DNA to focus on the individual [63]. Precision nutrition utilizes a series of individual characteristics, including sex/gender, as well as omics and genetic markers, to make more personalized nutrition recommendations [64]. This approach could be a suitable solution for providing nutritional recommendations to transgender individuals, concentrating on individual needs rather than generalizations. Precision nutrition presents an opportunity to provide holistic care and should factor in social determinants of health that affect the transgender community, such as social stigma and minority stress. However, personalized nutrition still requires guidelines or reference values to be able to personalize the patient’s diet and achieve optimal nutritional status [63].

Nevertheless, the lack of conclusive nutritional data highlights that having an evidence-based dietary guideline for transgender individuals is especially pertinent for clinicians to be able to make the appropriate recommendations for daily intake levels of essential nutrients and energy required for weight management [7,55,61]. In order to create evidence-based clinical practice guidelines, there needs to be more robust data on the unique nutritional needs of transgender individuals, which should also take into account a patient’s use of hormonal and/or surgical treatment [9].

## Healthcare provider competencies and discrimination

Research suggests that LGBTQ youth include nutrition as an important health concern they would like to discuss with providers [65]. Many LGBTQ people have reported experiencing stigma and discrimination when accessing health services, leading some individuals to delay necessary health care or forego it altogether [65]. Alarmingly, 50% of transgender individuals report having to teach their medical providers about transgender care [66]. Moreover, uninformed professionals may make it difficult for providers to create rapport with clients. A critical challenge involved in encouraging access to health and nutrition support includes lack of culturally-competent treatment, poor awareness of identity issues, and insufficient eating disorders education among providers, such as Registered Dietitian Nutritionists, leading to a lack of confidence [5].

This lack of provider knowledge highlights the need for research aimed at exploring limitations in education practices related to LGBTQ+ care and has important implications for public health nutrition. In order to help address the specific health care and nutrition needs of the LGBTQ+ community in an inclusive and affirming manner moving forward, medical provider training curricula must implement LGBTQ-related programming, with a focus on explicit and implicit bias reduction interventions [67], queer pedagogical practices [68], and health equity and intersectionality [69].

Despite major societal advancements over the past few decades for the LGBTQ+ community, there is a substantial lack of research and data on LGBTQ+ health, particularly within the context of eating behaviors and nutrition. We found several LGBTQ+ nutrition considerations of particular concern, including vulnerability to economic and food insecurity, obesity risk among individuals who identify as transgender, and subsequent bidirectional impacts of mental health outcomes such as societal discrimination, eating disorders, weight management, and provider competencies (Figure 1). Areas of nutrition disparities among individuals who identify as LGBTQ+ lacking substantial research include personalized and precision nutrition, social and behavioral determinants of health, quality of diet, body image, and clinician cultural competency and responsiveness. Additional gaps in knowledge include evidence-based dietary guidelines and clinician recommendations for the potential unique nutritional and weight management needs of people who identify as transgender. A key future research direction should include how supportive nutrition actions and interventions may impact weight management and overall quality of life among transgender populations who have elected to undergo gender-affirming treatments. Furthermore, there is a strong need for studies examining within-group and intersectional differences among LGBTQ+ populations. Future investigators should also prioritize robust methodologies, such as randomized control trials and clinical nutrition translation and implementation, to help address health disparities within sexual minority subgroups. Notably, nutrition actions/interventions and provider recommendations aimed toward the LGBTQ+ community must be culturally responsive in nature.

In summary, there is limited literature on dietary concerns and, consequently, nutrition care guidelines for LGBTQ+, which is a major gap in the nutrition field. This highlights the opportunity for public health practitioners to translate nutrition science into specific food and nutrient recommendations that are tailored to LGBTQ+ specific lifestyle, life cycle, and medical needs, as well as implement nutrition strategies based on culture, ethnicity, and income status, among other social implications. Considering the known detrimental impacts of the COVID-19 pandemic on both overall nutrition and dietary patterns as a whole and LGBTQ+ health, additional studies are critical to determine appropriate and effective nutrition guidelines and recommendations for the LGBTQ+ community during public health emergencies.

## Acknowledgements

The authors appreciate the comments from our colleagues from the 2022 Introduction to Nutrition in Public Health course at Harvard T.H. Chan School of Public Health. The insights and guidance from Dr. S. Bryn Austin and from members of the LGBTQ+ community are highly valued.

## Author contributions

The authors’ responsibilities were as follows – EMF, AGY, SC, SG: conceptualized the topic, researched and analyzed the background literature, and drafted the manuscript; KM, SNB, JM: provided substantial scholarly guidance on the conception of the topic and manuscript draft and interpretation; EMF, AGY, KM, SNB, JM: provided critical review, commentary, and revisions to the manuscript, All authors accept primary responsibility for the final content, and all authors: read and approved the final manuscript.

## Conflict of interest

The authors report no conflicts of interest.

## Funding

The authors reported no funding received for this study.
